# The complete mitochondrial genome sequence of *Asclepios apicalis* (Gerromorpha: Gerridae)

**DOI:** 10.1080/23802359.2021.1909436

**Published:** 2021-04-22

**Authors:** Yuxia Liu, Hongxia Yang, Xiaoxuan Tian, Ying Cui

**Affiliations:** State Key Laboratory of Component-Based Chinese Medicine, Tianjin University of Traditional Chinese Medicine, Tianjin, China

**Keywords:** *Asclepios apicalis*, mitochondrial genome

## Abstract

In this study, the complete mitochondrial genome of *Asclepios apicalis* was sequenced and assembled, which was first reported in *Asclepios*. The mitogenome of *Asclepios apicalis* was 15,391 bp in length, and it contained 22 transfer RNA (tRNA) genes, two ribosomal RNA (rRNA) genes, 13 protein-coding genes (PCGs), and a control region (D-loop), the overall base nucleotide compositions encoded was 42.9% A, 14.3% C, 10.0% G, and 32.8% T.

*Asclepios apicalis* (Esaki 1924) is a water bug that belongs to the family Gerridae. The water bugs conquered water surfaces worldwide and diversified to occupy ponds, streams, lakes, and even oceans. Their hairs allow them to maintain their body weight at the surface of the water and protect them from moisture and drowning (Finet et al. 2018). In addition, the legs of these insects are equipped with various grooming combs that contribute to cleaning and tidying the hair layers so as to obtain optimal functional efficiency. Water bugs usually play an important role in freshwater ecosystems, and information about them is essential for the study of water biology and the proper management of aquatic habitats (Cordeiro and Moreira 2015; Havemann et al. 2018). However, there is still no study on the complete mitogenome sequences for *A. apicalis*. Hence, it is crucial to determine the whole mitogenome of *A. apicalis* for further study.

The specimen of *A. apicalis* was collected from Kunming city, Yunnan Province (102.73°E, 25.04°N), China. The voucher specimen was deposited in Institute of Entomology, College of Life Sciences, Nankai University (Ying Cui, CQL8179270@126.com), under the voucher number of A20060702. The collected specimen was preserved in 95% ethanol at −20 °C until DNA extraction. Total genomic DNA was extracted from muscle tissue of the specimen using the CTAB method (Reineke et al. 1998). Polymerase chain reactions (PCRs) were performed with TaKaRa LA PCR Kit Ver. 2.1 following the manufacturer's recommendations. The PCR products of the Gel electrophoresis were purified in 0.7% agarose gel and then both strands were sequenced with primer walking by Beijing Sunbiotech Co. Ltd. (Beijing, China). BioEdit 7.0 was used for sequence alignment and assembly. The characters of base composition and distribution were analyzed using Geneious Prime 2020 software (Kearse et al. 2012). The mitogenome was annotated using MITOS web server (Yu et al. 2019), and was manually checked and adjusted the annotation using *Aquarius paludum* (GenBank accession number: NC012841) as the reference sequence. The online tRNAscan-SE search server (Lowe and Chan 2016) was used to annotate the transfer RNA (tRNA) gene to determine its position, and the parameter settings were default. The start and stop codons of protein-coding genes (PCGs) were manually adjusted to fit open reading frames. The maximum-likelihood (ML) methods were used to construct the phylogenetic tree. The ML trees were obtained with 1000 bootstrap replications using MEGA X (Kumar et al. 2018).

The results revealed that the total length of mitogenome of *A. apicalis* was 15,391 bp, with the base composition of 42.9% for A, 14.3% for C, 10.0% for G, 32.8% for T, and with a high A + T content of 75.7%. The length of the non-coding region was 803 bp, which accounts for 5.2% of the total length. The length of the coding region was 14,588 bp and it contained 37 coding genes (22 tRNA genes, two ribosomal RNA (rRNA) genes, and 13 PCGs).

To assess the phylogenetic position of *A. apicalis*, the ML phylogenetic tree was constructed using the complete mitogenome sequence of Gerridae. The complete mitogenome sequence of *Hydrometra greeni* was set as the outgroup (Hua et al. 2009). The results showed that *A. apicalis* formed an independent lineage in the family Gerridae (Figure 1). Overall, the complete mitogenome of *A. apicalis* can contribute to further phylogenetic study within Gerridae.

**Figure 1.**
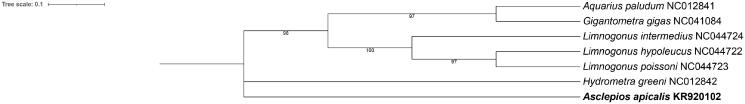
Maximum-likelihood (ML) tree inferred from complete mitochondrial genome sequences of Gerridae. The *Hydrometra greeni* was set as the outgroup. GenBank accession numbers for all sequences are listed in the figure. The numbers above the lines were bootstrap support values for ML analyses.

## Data Availability

The data that support the findings of this study is openly available in GenBank at https://www.ncbi.nlm.nih.gov/genbank/, reference number KR920102. The raw data of sequencing are openly available in Figshare at https://figshare.com/s/b7a0b835184c0e161da3. Acknowledgements We thank Dr Wenjun Bu (Nankai University) for the help of providing materials and sequencing. This project was supported by the Science & Technology Development Fund of Tianjin Education Commission for Higher Education (Project No. 2018KJ009).
